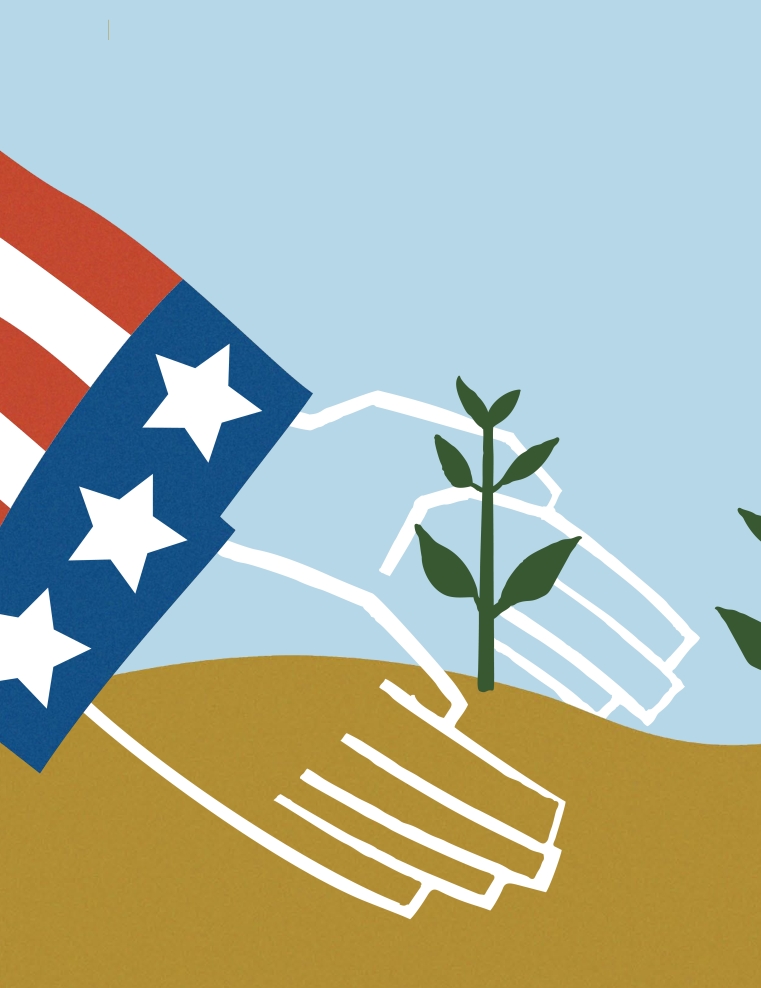# The New USDA: Cultivating Change

**DOI:** 10.1289/ehp.117-a402

**Published:** 2009-09

**Authors:** David A. Taylor

**Affiliations:** **David A. Taylor** writes for *The Washington Post* and *Smithsonian* and is the author of *Ginseng, the Divine Root*, about the science and subculture surrounding the medicinal plant. He teaches science writing at The Writer’s Center in Maryland

In proclaiming the week of August 23–29 National Community Gardening Week, Agriculture Secretary Tom Vilsack noted, “Community gardens provide numerous benefits including opportunities for local food production, resource conservation, and neighborhood beautification. But they also promote family and community interaction and enhance opportunities to eat healthy, nutritious foods. Each of these benefits is something we can and should strive for.”

Vilsack’s statement was the latest in a string of signals in the past 7 months that suggest significant changes are afoot at the U.S. Department of Agriculture (USDA). On 29 July 2009 Agriculture Deputy Secretary Kathleen Merrigan inaugurated a new rooftop garden at the offices of the USDA Economic Research Service. In early spring, Vilsack “broke pavement” for a vegetable garden known as the People’s Garden, which was planted in front of USDA headquarters across from the National Mall. And a March 2009 planning meeting to discuss the People’s Garden and other sustainability initiatives, chaired by Vilsack, included people not typically seen at USDA meetings in the past: representatives from community garden associations, local food policy councils, botanical gardens, and the Rodale Institute, a nonprofit organization in Pennsylvania dedicated to organic farming research. Vilsack “talked about sustainability, linking agriculture, food, and human health in a way that you haven’t heard [from USDA],” recalls Rose Hayden-Smith, a fellow at the Minneapolis-based nonprofit Institute for Agriculture and Trade Policy and master gardener for the California Cooperative Extension service.

## Community Gardening Gets a Head Gardener?

The aim of the People’s Garden is for the USDA to serve as a model for better nutritional, community, and environmental health. In the publicity around the People’s Garden, Vilsack noted several health benefits of community gardening, including increased access to affordable fresh produce and more exercise. Small, preliminary studies further suggest that indirect community health benefits may range from reduced crime (the “Whitmire Study” conducted in St. Louis, Missouri, by a local community group and available at www.gatewaygreening.org) to increased property values (Been and Voicu, *New York University Law and Economics Working Papers*, Paper 46 [2006]). Several studies published in the July–September 2005 issue of *HortTechnology*, the journal of The American Society for Horticultural Science, also linked student gardens to better achievement in science. In just about all cases public gardens increase community interaction and provide intergenerational learning opportunities, says Bill Maynard, vice president of the nonprofit American Community Garden Association.

In cities, environmental benefits of community gardens, “green roofs,” and other vegetated spaces include reduced heat island effects because gardens absorb and radiate less heat than pavement (see, for example, research by Atsuko Nonomura et al. published online 3 June 2009 ahead of print in the *Journal of Environmental Management*) and reduced stormwater runoff because gardens absorb rain (see, for example, research by Nicholaus D. VanWoert et al. in the 11 May 2005 issue of the *Journal of Environmental Quality*). Public gardens can involve relatively high up-front costs, says Maynard, but after they are constructed they can be sustained by dues and volunteer labor.

The People’s Garden itself incorporates a number of sustainable agriculture principles. Crops are planted for seasonal variety, from cool-weather lettuce and field peas to summer tomatoes, squash, and herbs, and less familiar cover crops—such as bearberry and sweet-spire—which are grown alongside food crops to help maintain soil fertility, retain moisture, and control weeds and pests. The garden will use minimal chemical inputs, and its soil has been enriched with organic compost.

But some observers see the USDA garden plot as a publicity gesture for an agency still fundamentally concerned with promoting big agriculture. “It’s a good symbol,” says Doug Gurian-Sherman, senior scientist on food and environment for the Union of Concerned Scientists (UCS), “but in terms of policy, it’s too early to say.”

## Changes in Names and More

Changes at the USDA began even before the new administration arrived. In October 2008, the federal Food Stamp Program was renamed the Supplemental Nutrition Assistance Program (SNAP) in part to include “nutrition” in the title and to clarify through the term “supplemental” that these funds were not meant to be the sole funding for food purchases in a household. On 1 October 2009, the USDA Cooperative State Research, Education, and Extension Service will be renamed the National Institute of Food and Agriculture, to bolster a wider range of research on food and farming methods, alternative fuels and efficiency.

For Hayden-Smith, the changes signal an awareness of agriculture’s broader importance beyond crops and commodities: “This is really a holistic view of food systems,” she says. More recently, under the 2009 economic stimulus package’s allocation for “green jobs,” the USDA has entertained proposals for funds to support training for gardening work in urban neighborhoods.

The USDA plays a major role as a provider of food assistance to low-income families through its nutrition assistance programs, which besides SNAP include the Women, Infants, and Children (WIC) Program for low-income women and their young children, the School Lunch and Breakfast Program, and international food aid. In those roles, says Hayden-Smith, the USDA has a huge footprint. “More than any other federal agency,” she says, “USDA affects our daily life.”

The USDA’s policy framework is shaped by the Farm Bill, a mammoth piece of omnibus legislation that comes up every 5 years. In health policy, USDA’s role has hinged largely on its assistance in developing the U.S. Dietary Guidelines for Americans, which it coordinates with the Department of Health and Human Services. In revising the guidelines every 5 years, the 2 departments alternate on taking the administrative lead; for the 2010 revision, the USDA has the lead role.

The revision process follows the Federal Advisory Committee Act rules, which require public comments on proposed changes. But some experts consider that overseeing the development of the dietary guidelines creates something of a conflict for the department. “USDA has the job of supporting U.S. agriculture at the same time as it sets U.S. nutrition policy,” explains Amy Lanou, an assistant professor who teaches food policy and nutrition politics at the University of North Carolina at Asheville. “Sometimes these two are at odds.”

Specifically, Lanou notes the dietary guidelines can fall short of optimal nutritional guidance, perhaps out of concern for adverse effects on agribusiness interests. For example, she says, in 2009 the USDA made a large purchase of dried milk to be distributed in food programs, in part to prop up sagging milk prices. Yet demographic data suggest an estimated 25% of U.S. children may be lactose-intolerant, says Lanou.

“My feeling is that if one in four children are likely to feel sick after drinking the milk provided to them at school through federally funded programs, and our USDA—instead of making an effort to provide other calcium-rich foods such as beans, greens, and calcium-fortified nondairy milks—is finding ways to distribute more milk and milk products into children, they are doing our kids, especially often our more disadvantaged kids, a real disservice,” Lanous says. She adds, “More milk, butter, and macaroni and cheese are not likely to be the solution to the childhood obesity problem.”

Hayden-Smith is more blunt: “We’ve got to get the [nutrition assistance] programs out of USDA,” she says. “It’s fundamentally a conflict of interest.”

Post acknowledges those concerns but explains, “This is an open and transparent process; comments from the public may be submitted during the two years the advisory committee meets and prepares its report, which are available for public viewing. And the current revision process employs an evidence-based approach, which is the gold standard for minimizing bias.”

Prior to the October 2008 start of the 2010 revision process the USDA created a Nutrition Evidence Library to facilitate the systematic, objective review of the scientific literature and to inform the Dietary Guidelines Advisory Committee. The library staff uses an electronic system to manage the process of systematically reviewing, summarizing, and assessing the quality of published research that is ultimately analyzed by the Advisory Committee members.

## Education through Inspiration

For an historical model of how sustainability can inform federal policy, Hayden-Smith points to World War I, when Herbert Hoover headed the U.S. Food Administration. In this role Hoover doubled U.S. food shipments to European allies and armies even without mandatory rationing at home simply by inspiring Americans to conserve, substitute, and produce their own food. The administration also fostered local food policy councils and taught young people about food with a nationwide school curriculum in food and farming.

Today, programs such as Future Farmers of America and 4-H help bring agricultural education to schools. The American Farm Bureau Federation (AFBF), an agriculture association, also helps fund a program called Ag in the Classroom that provides classroom materials to teach both rural and urban students about agriculture. “Most Americans, including teachers, are multiple generations removed from the farm, and the program is designed to be sure that teachers have correct information instead of myths to pass along to students,” says Tara Smith, congressional relations director at the AFBF. “For example, chocolate milk does not come from brown cows, [but] you would be surprised how many people think that it does.”

Hayden-Smith would welcome more such school programs. “The most important single federal policy, I think, would be to create and mandate a federal curriculum [that teaches students those fundamentals],” she says.

Does that fit with how USDA sees its role now? “It does,” says Robert Post, deputy director of the Center for Nutrition Policy and Promotion, the USDA’s lead agency for nutrition policy. “One of the goals given to us is to strive for better coordination with the Department of Education,” including weaving farm-to-table education into school curricula.

According to Post, the links between farming and nutrition have never had a higher profile. Within days of joining the USDA, Vilsack identified priorities for his agencies, says Post, including fighting the obesity epidemic. “That elevates quite visibly all the efforts related to nutrition at USDA.”

## Farm Bill: A Mixed Bag

The Farm Bill of 2008 was controversial for continuing farm subsidies for commodity crops including corn, cotton, rice, wheat, and peanuts. The persistence of commodity subsidies dismays Jeff Moyer, farm director at the Rodale Institute and chairman of the USDA National Organic Standards Board, who called them “a disservice to the American taxpayer and to some extent to the farmers.” Rodale isn’t against payments to farmers, says Moyer, but the institute would rather see payments based on criteria such as improvements in soil fertility, reduction of erosion and chemical residues, increased soil capacity to store carbon. For Moyer, commodity subsidy payments signal a priority for cheap trade items rather than long-term stewardship. Still, he calls the 2008 Farm Bill, with its allocation of funds for sustainable agriculture and soil conservation, “a step in the right direction.”

Gurian-Sherman sees promise in some of Vilsack’s staff choices, including Merrigan, who is the former director of the Agriculture, Food and Environment program at Tufts University. He says Merrigan has “a history of working on and being sympathetic to sustainable agriculture issues.”

On the other hand, he objects to a proposed rule that is intended to consolidate regulation of genetically engineered plants under the Plant Protection Act. Gurian-Sherman says the current version of the rule—which has not been finalized—would weaken regulation of genetically engineered plants. Such plants could be approved for commercial use by the USDA Animal and Plant Health Inspection Service unless they meet the narrowly defined criteria for a “noxious weed”; that is, that they cause extreme environmental harm such as completely overruning their surroundings. This could leave most genetically engineered plants virtually unregulated, even if they present environmental or public health concerns separate from those of noxious weeds. “From our perspective,” he says, “the biotech industry hasn’t been properly regulated, and this is a move in the wrong direction.”

Besides tougher rules for biotechnology, Gurian-Sherman recommends more studies by the USDA Agricultural Research Service on organic and low-input farming methods, long-term crop rotation, and cover crops that increase carbon sequestration. He, too, recommends fewer subsidies for commodity crops. When the Farm Bill is back on the table in a few years, says Gurian-Sherman, there will be a chance to re-examine the subsidy system. Like Moyer, he’s not against subsidies per se, but he believes payments should promote a longer-term view of resource stewardship and sustainable farming.

The growing number of large concentrated animal feeding operations (CAFOs) also concerns Gurian-Sherman. According to the latest USDA Census of Agriculture, released in February 2009, the vast majority of hogs, turkeys, and chickens sold in 2007 were raised on CAFOs. To give smaller producers fair access in the processing industry, UCS urges stricter enforcement of the amended Packers and Stockyard Act, which provides anti-trust protections designed to maintain competition in the livestock industry. According to advocacy groups such as the Institute for Local Self-Reliance, such enforcement is even more important if a small number of companies control a large percentage of the market. Opening USDA nutrition assistance programs to permit local substitutions for department-purchased food items could be one way to level the field for smaller producers, says Gurian-Sherman.

Hayden-Smith agrees that such a change could significantly affect how local and state governments purchase food, adding, “The purchasing power of state and local governments, institutions, and agencies is huge.” Indeed, with an annual budget that rose to $133 billion in fiscal year 2010, the USDA ranks among only a handful of departments that saw its funding increase amid an economic recession. Two-thirds of the USDA’s budget goes to nutrition assistance programs, according to the USDA’s *FY2010 Budget Summary and Annual Performance Plan*.

As an institutional purchaser, the USDA exerts significant influence, yet produce from school gardens heretofore has not been permitted in school lunch programs. Lanou suggests that produce from school gardens, being locally grown, may be more sustainable (because of lower transport costs) and fresher than USDA-purchased foods trucked in from farther away.

Post says, “There should be more fruits and vegetables in school lunch programs and more accessibility to farmers’ markets.” The recent addition of vouchers for fresh produce to WIC food allotments has important educational value—teaching recipients about nutrition—as well as economic impact, says Lanou.

A First Family that raises fruits and vegetables—as the Obama household is doing—also could very well help in this endeavor. It is educationally and nutritionally important, Lanou says, when Michelle Obama tells schoolchildren they have the power to grow their own food and shows them its nutritional value. Adds Hayden-Smith, “Gardening is the gateway [that leads] to interest in the food system.” In other words: Get kids interested in growing vegetables, and they’re on the path to healthier eating.

## The Farmers’ Perspective

Farm industry groups familiar with Vilsack when he was Iowa’s governor have not embraced all the changes in the USDA. “USDA and this administration have made it abundantly clear what their priorities are, and it isn’t production agriculture,” says Smith. “Not everybody has access to a garden or land to grow a garden. We just want to make sure the public maintains respect for [large-scale] agriculture.”

Yet the AFBF does support farmers’ markets and organic farming, says Smith: “Our farmers respond to where the demand is.”

Another piece of legislation may complicate or improve the USDA’s position with farmers, depending on who’s talking: the American Clean Energy and Security Act of 2009 (also known as the Waxman-Markey bill), which was passed by the House in June and currently awaits Senate vote. The AFBF saw the bill as penalizing farmers in the way it proposed integrating carbon surcharges into farmers’ costs for fuel and other inputs. By the AFBF’s calculation, the proposal would raise farmers’ costs by 10%, yet farm produce prices are relatively static. Says Smith, “It’s a negative for our farmers.”

For Moyer, however, a top policy priority is improving ways of putting dollar values on carbon storage in the soil and getting those methods—and other methods for valuing ecosystem services—into the next Farm Bill. Moyer believes all farmers would ultimately benefit from a system that rewards stewardship of the soil. “If we can take carbon out of the air [and reduce its greenhouse impact],” he says, “that’s good for everybody.” He adds, “The soil holds the future for all of us. We need to manage it with that long-term view in mind.”

## Figures and Tables

**Figure:**